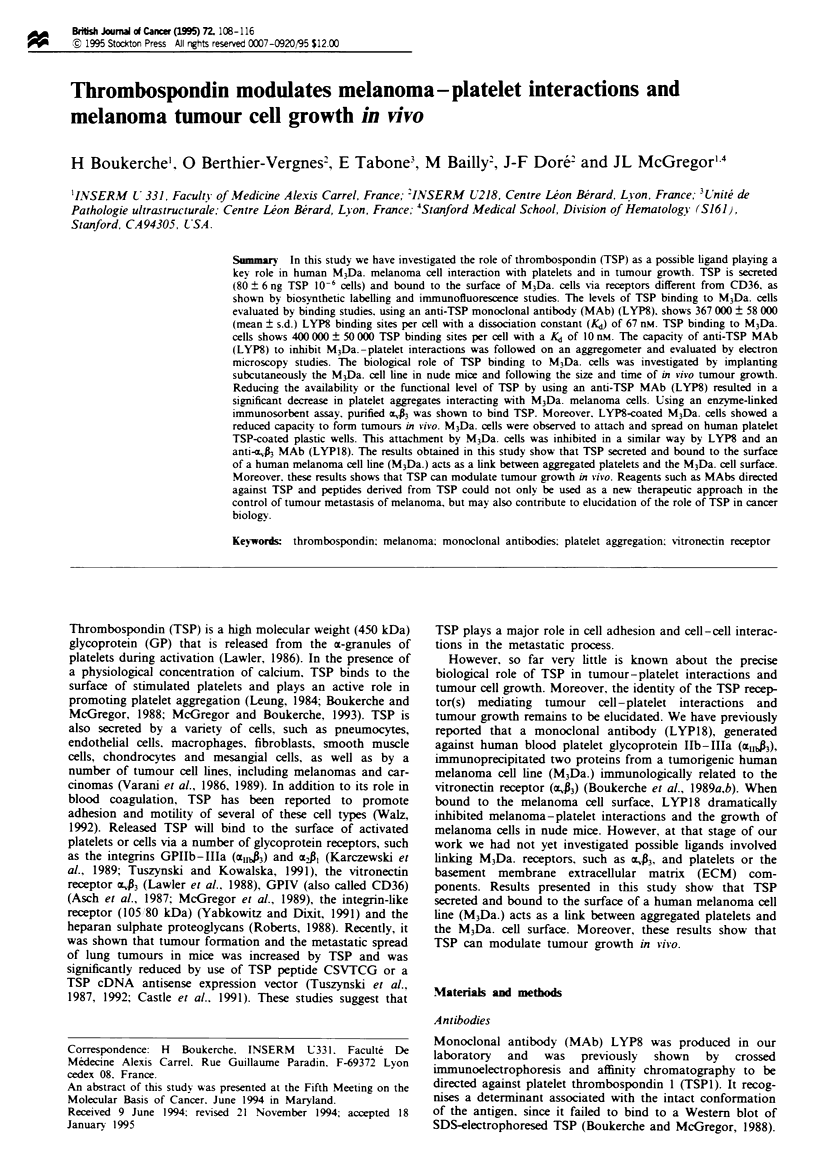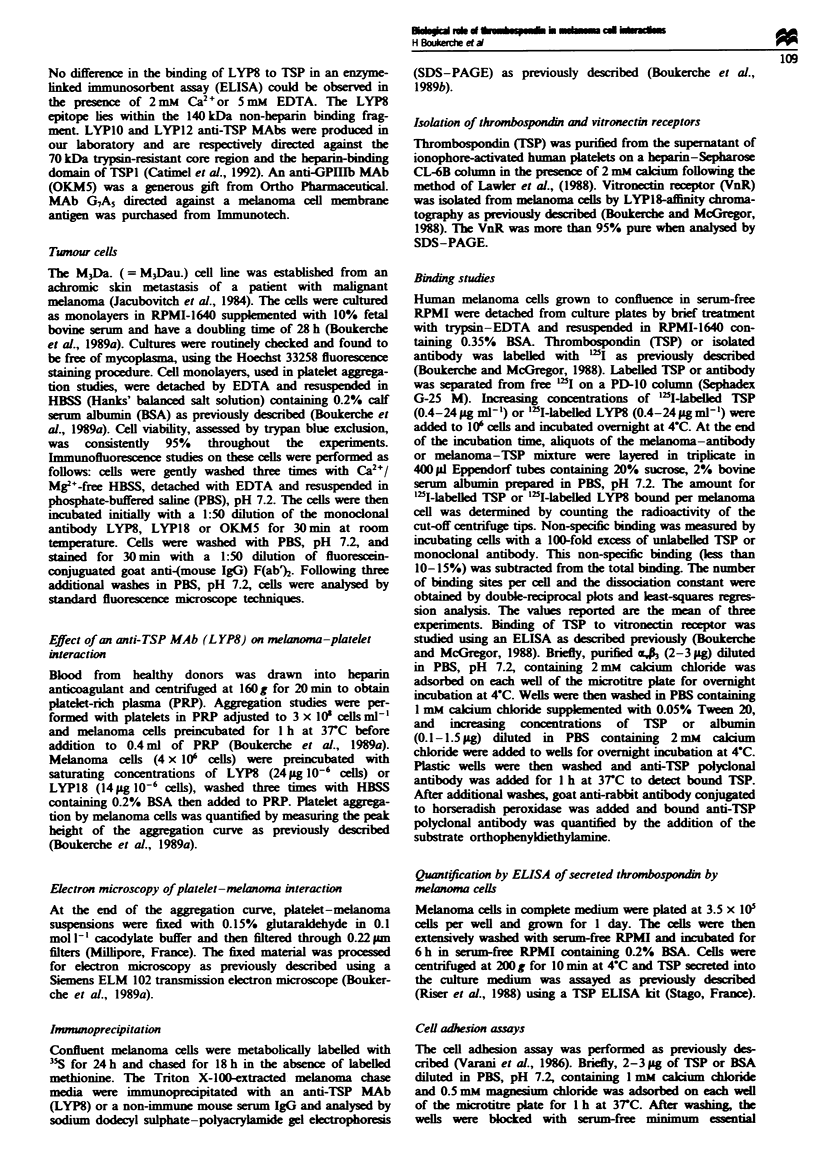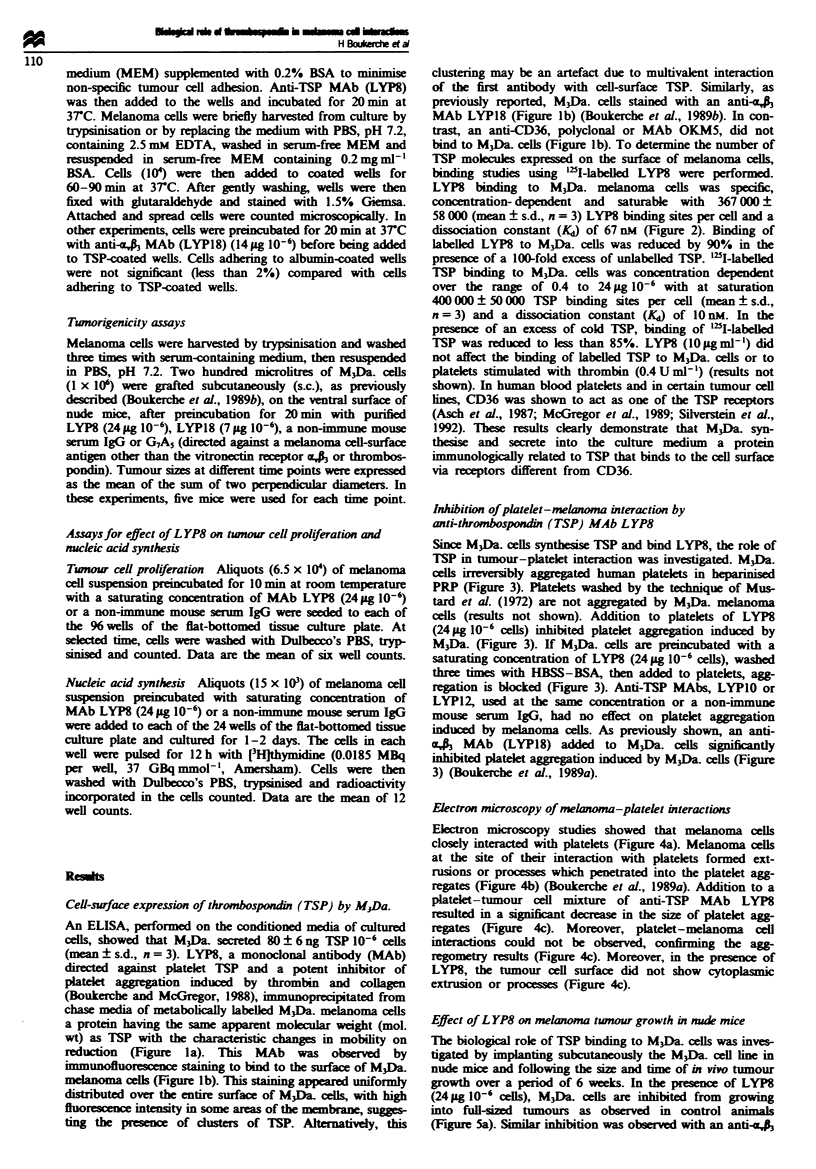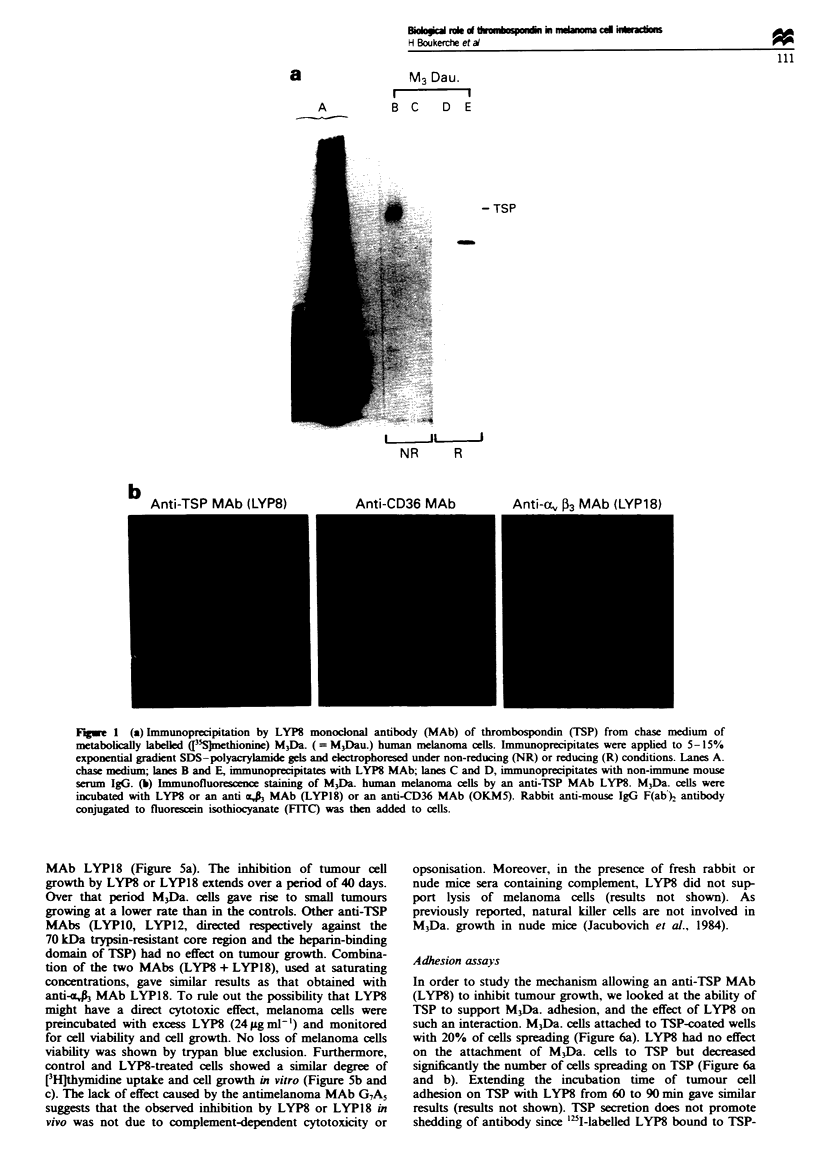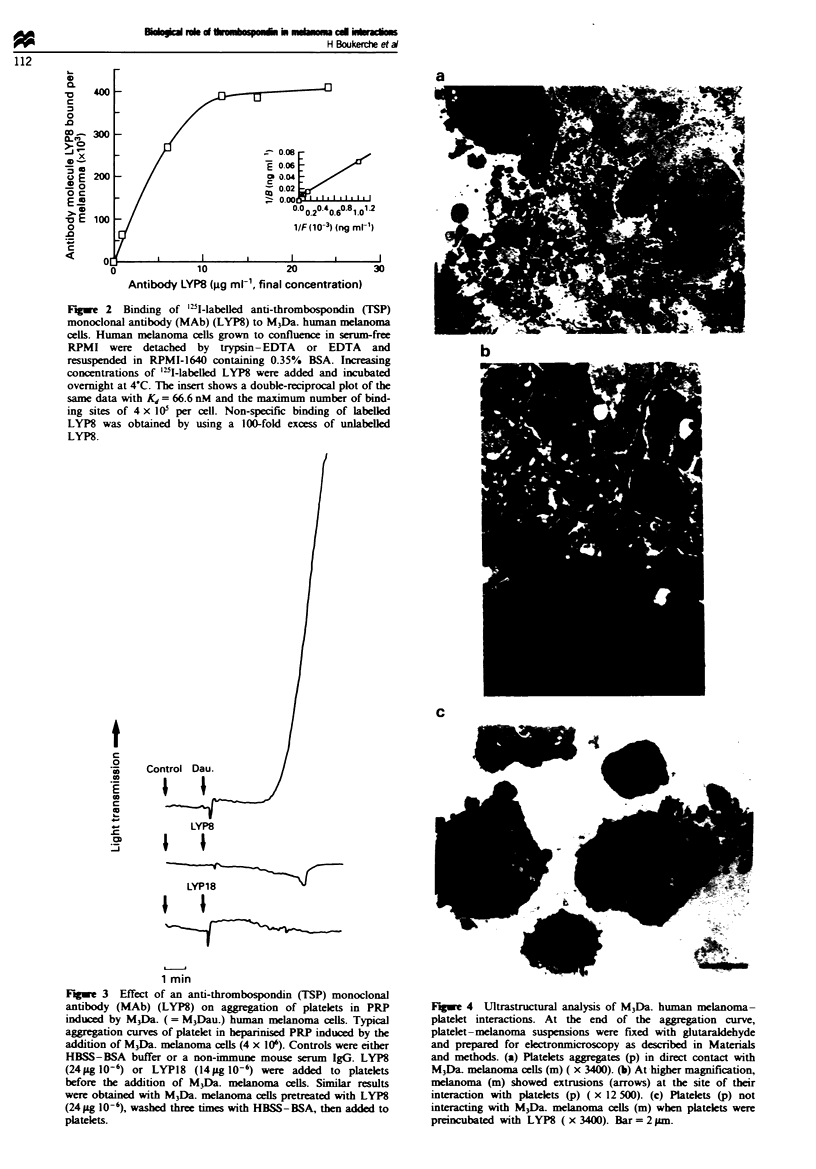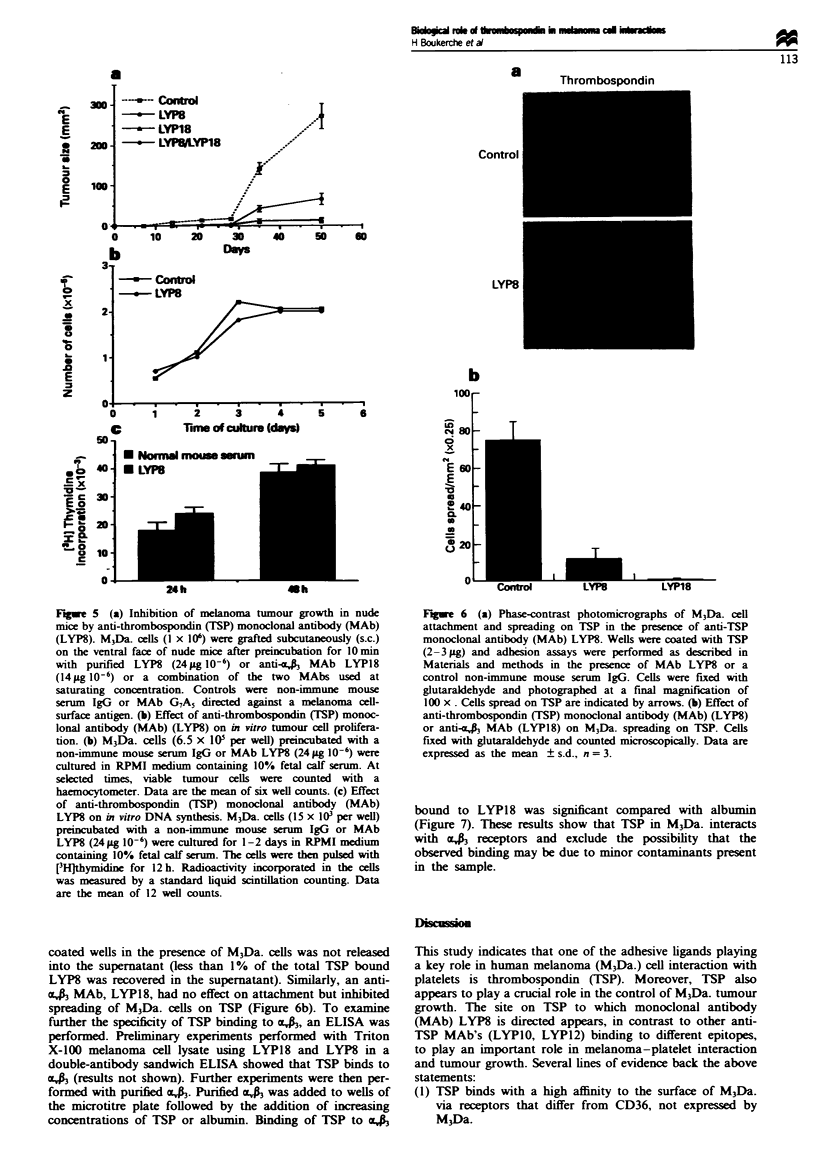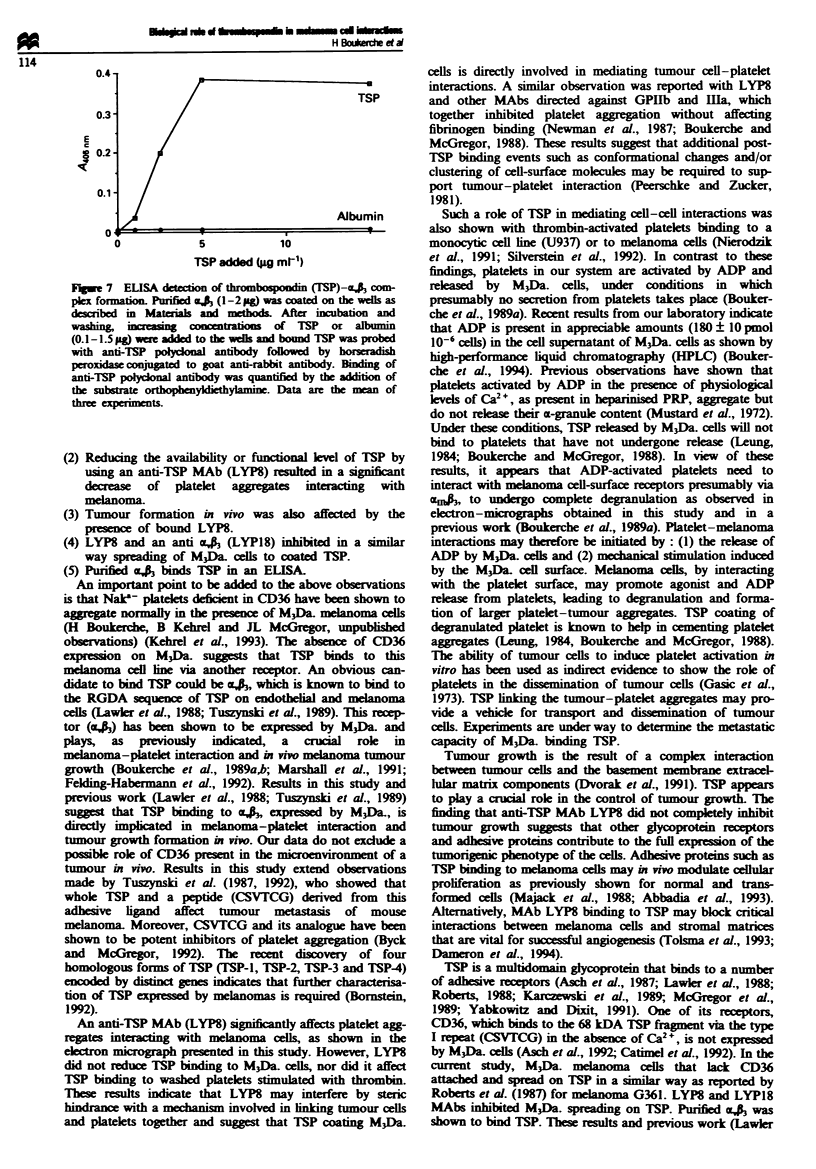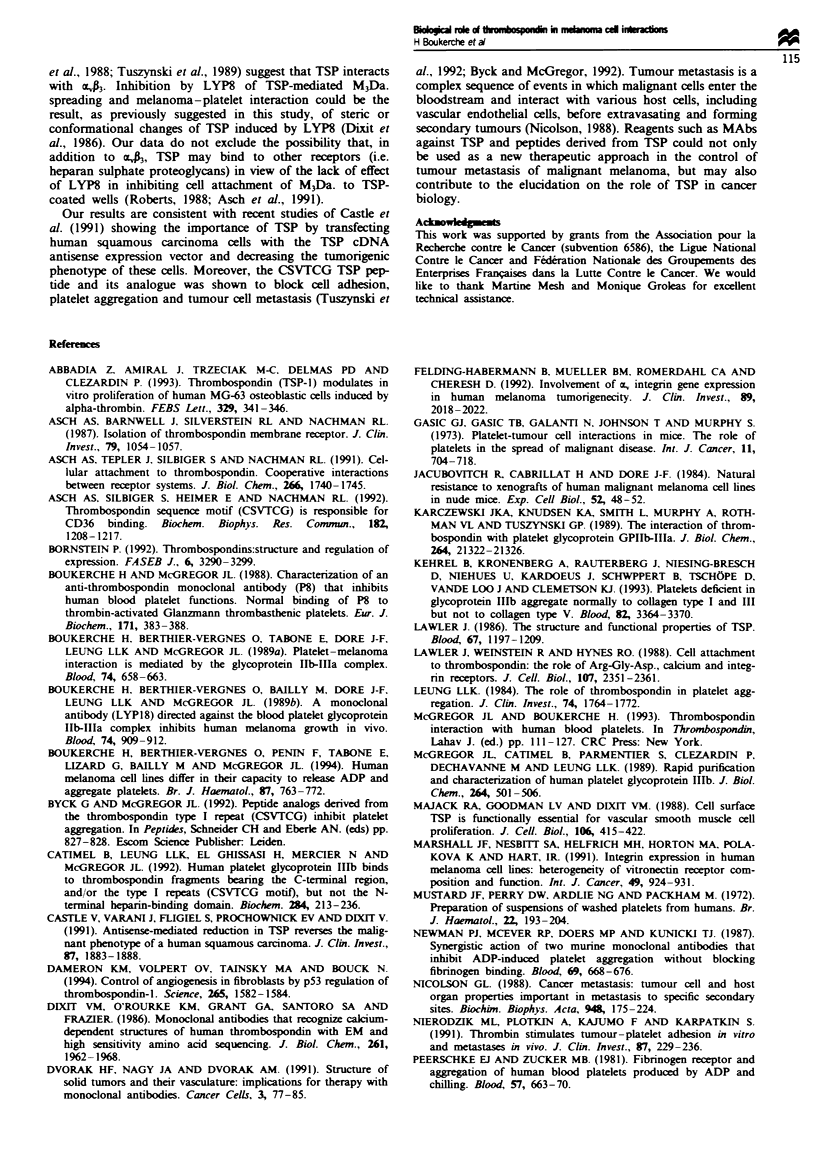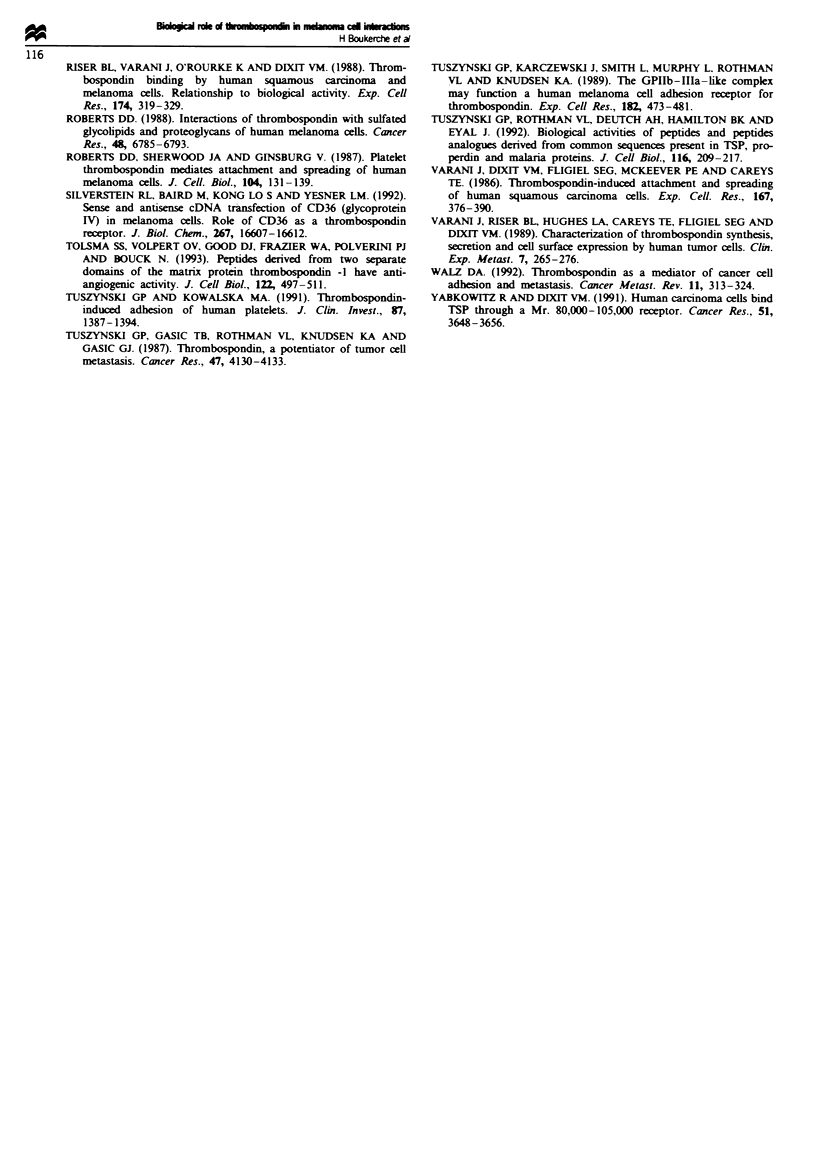# Thrombospondin modulates melanoma--platelet interactions and melanoma tumour cell growth in vivo.

**DOI:** 10.1038/bjc.1995.285

**Published:** 1995-07

**Authors:** H. Boukerche, O. Berthier-Vergnes, E. Tabone, M. Bailly, J. F. Doré, J. L. McGregor

**Affiliations:** INSERM U 331, Faculty of Medicine Alexis Carrel, France.

## Abstract

**Images:**


British Journal of Cancer (1995) 72, 108-116

© 1995 Stockton Press All rights reserved 0007-0920/95 $12.00

### Thrombospondin modulates melanoma–platelet interactions and melanoma tumour cell growth *in vivo*

H Boukerche[1], O Berthier-Vergnes[2], E Tabone[3], M Bailly[2], J-F Doré[2] and JL McGregor[1,4]

[1]*INSERM U 331, Faculty of Medicine Alexis Carrel, France;* [2]*INSERM U218, Centre Léon Bérard, Lyon, France;* [3]*Unité de Pathologie ultrastructurale; Centre Léon Bérard, Lyon, France;* [4]*Stanford Medical School, Division of Hematology (S161), Stanford, CA94305, USA.*

**Summary** In this study we have investigated the role of thrombospondin (TSP) as a possible ligand playing a key role in human $M_3Da.$ melanoma cell interaction with platelets and in tumour growth. TSP is secreted ($80 \pm 6$ ng TSP $10^{-6}$ cells) and bound to the surface of $M_3Da.$ cells via receptors different from CD36, as shown by biosynthetic labelling and immunofluorescence studies. The levels of TSP binding to $M_3Da.$ cells evaluated by binding studies, using an anti-TSP monoclonal antibody (MAb) (LYP8), shows $367\,000 \pm 58\,000$ (mean ± s.d.) LYP8 binding sites per cell with a dissociation constant ($K_d$) of 67 nM. TSP binding to $M_3Da.$ cells shows $400\,000 \pm 50\,000$ TSP binding sites per cell with a $K_d$ of 10 nM. The capacity of anti-TSP MAb (LYP8) to inhibit $M_3Da.$-platelet interactions was followed on an aggregometer and evaluated by electron microscopy studies. The biological role of TSP binding to $M_3Da.$ cells was investigated by implanting subcutaneously the $M_3Da.$ cell line in nude mice and following the size and time of *in vivo* tumour growth. Reducing the availability or the functional level of TSP by using an anti-TSP MAb (LYP8) resulted in a significant decrease in platelet aggregates interacting with $M_3Da.$ melanoma cells. Using an enzyme-linked immunosorbent assay, purified $\alpha_v\beta_3$ was shown to bind TSP. Moreover, LYP8-coated $M_3Da.$ cells showed a reduced capacity to form tumours *in vivo*. $M_3Da.$ cells were observed to attach and spread on human platelet TSP-coated plastic wells. This attachment by $M_3Da.$ cells was inhibited in a similar way by LYP8 and an anti-$\alpha_v\beta_3$ MAb (LYP18). The results obtained in this study show that TSP secreted and bound to the surface of a human melanoma cell line ($M_3Da.$) acts as a link between aggregated platelets and the $M_3Da.$ cell surface. Moreover, these results shows that TSP can modulate tumour growth *in vivo*. Reagents such as MAbs directed against TSP and peptides derived from TSP could not only be used as a new therapeutic approach in the control of tumour metastasis of melanoma, but may also contribute to elucidation of the role of TSP in cancer biology.

**Keywords:** thrombospondin; melanoma; monoclonal antibodies; platelet aggregation; vitronectin receptor

Thrombospondin (TSP) is a high molecular weight (450 kDa) glycoprotein (GP) that is released from the α-granules of platelets during activation (Lawler, 1986). In the presence of a physiological concentration of calcium, TSP binds to the surface of stimulated platelets and plays an active role in promoting platelet aggregation (Leung, 1984; Boukerche and McGregor, 1988; McGregor and Boukerche, 1993). TSP is also secreted by a variety of cells, such as pneumocytes, endothelial cells, macrophages, fibroblasts, smooth muscle cells, chondrocytes and mesangial cells, as well as by a number of tumour cell lines, including melanomas and carcinomas (Varani *et al.*, 1986, 1989). In addition to its role in blood coagulation, TSP has been reported to promote adhesion and motility of several of these cell types (Walz, 1992). Released TSP will bind to the surface of activated platelets or cells via a number of glycoprotein receptors, such as the integrins GPIIb-IIIa ($\alpha_{IIb}\beta_3$) and $\alpha_2\beta_1$ (Karczewski *et al.*, 1989; Tuszynski and Kowalska, 1991), the vitronectin receptor $\alpha_v\beta_3$ (Lawler *et al.*, 1988), GPIV (also called CD36) (Asch *et al.*, 1987; McGregor *et al.*, 1989), the integrin-like receptor (105/80 kDa) (Yabkowitz and Dixit, 1991) and the heparan sulphate proteoglycans (Roberts, 1988). Recently, it was shown that tumour formation and the metastatic spread of lung tumours in mice was increased by TSP and was significantly reduced by use of TSP peptide CSVTCG or a TSP cDNA antisense expression vector (Tuszynski *et al.*, 1987, 1992; Castle *et al.*, 1991). These studies suggest that TSP plays a major role in cell adhesion and cell–cell interactions in the metastatic process.

However, so far very little is known about the precise biological role of TSP in tumour–platelet interactions and tumour cell growth. Moreover, the identity of the TSP receptor(s) mediating tumour cell–platelet interactions and tumour growth remains to be elucidated. We have previously reported that a monoclonal antibody (LYP18), generated against human blood platelet glycoprotein IIb–IIIa ($\alpha_{IIb}\beta_3$), immunoprecipitated two proteins from a tumorigenic human melanoma cell line ($M_3Da.$) immunologically related to the vitronectin receptor ($\alpha_v\beta_3$) (Boukerche *et al.*, 1989*a*,*b*). When bound to the melanoma cell surface, LYP18 dramatically inhibited melanoma–platelet interactions and the growth of melanoma cells in nude mice. However, at that stage of our work we had not yet investigated possible ligands involved linking $M_3Da.$ receptors, such as $\alpha_v\beta_3$, and platelets or the basement membrane extracellular matrix (ECM) components. Results presented in this study show that TSP secreted and bound to the surface of a human melanoma cell line ($M_3Da.$) acts as a link between aggregated platelets and the $M_3Da.$ cell surface. Moreover, these results show that TSP can modulate tumour growth *in vivo*.

### Materials and methods

### Antibodies

Monoclonal antibody (MAb) LYP8 was produced in our laboratory and was previously shown by crossed immunoelectrophoresis and affinity chromatography to be directed against platelet thrombospondin 1 (TSP1). It recognises a determinant associated with the intact conformation of the antigen, since it failed to bind to a Western blot of SDS-electrophoresed TSP (Boukerche and McGregor, 1988).

Correspondence: H Boukerche, INSERM U331, Faculté De Médecine Alexis Carrel, Rue Guillaume Paradin, F-69372 Lyon cedex 08, France.
An abstract of this study was presented at the Fifth Meeting on the Molecular Basis of Cancer, June 1994 in Maryland.
Received 9 June 1994; revised 21 November 1994; accepted 18 January 1995

No difference in the binding of LYP8 to TSP in an enzyme-linked immunosorbent assay (ELISA) could be observed in the presence of 2 mM $Ca^{2+}$ or 5 mM EDTA. The LYP8 epitope lies within the 140 kDa non-heparin binding fragment. LYP10 and LYP12 anti-TSP MAbs were produced in our laboratory and are respectively directed against the 70 kDa trypsin-resistant core region and the heparin-binding domain of TSP1 (Catimel *et al.*, 1992). An anti-GPIIIb MAb (OKM5) was a generous gift from Ortho Pharmaceutical. MAb $G_7A_5$ directed against a melanoma cell membrane antigen was purchased from Immunotech.

*Tumour cells*

The $M_3Da$. ( = $M_3Dau$.) cell line was established from an achromic skin metastasis of a patient with malignant melanoma (Jacubovitch *et al.*, 1984). The cells were cultured as monolayers in RPMI-1640 supplemented with 10% fetal bovine serum and have a doubling time of 28 h (Boukerche *et al.*, 1989*a*). Cultures were routinely checked and found to be free of mycoplasma, using the Hoechst 33258 fluorescence staining procedure. Cell monolayers, used in platelet aggregation studies, were detached by EDTA and resuspended in HBSS (Hanks' balanced salt solution) containing 0.2% calf serum albumin (BSA) as previously described (Boukerche *et al.*, 1989*a*). Cell viability, assessed by trypan blue exclusion, was consistently 95% throughout the experiments. Immunofluorescence studies on these cells were performed as follows: cells were gently washed three times with $Ca^{2+}$/$Mg^{2+}$-free HBSS, detached with EDTA and resuspended in phosphate-buffered saline (PBS), pH 7.2. The cells were then incubated initially with a 1:50 dilution of the monoclonal antibody LYP8, LYP18 or OKM5 for 30 min at room temperature. Cells were washed with PBS, pH 7.2, and stained for 30 min with a 1:50 dilution of fluorescein-conjuguated goat anti-(mouse IgG) $F(ab')_2$. Following three additional washes in PBS, pH 7.2, cells were analysed by standard fluorescence microscope techniques.

*Effect of an anti-TSP MAb (LYP8) on melanoma-platelet interaction*

Blood from healthy donors was drawn into heparin anticoagulant and centrifuged at 160 *g* for 20 min to obtain platelet-rich plasma (PRP). Aggregation studies were performed with platelets in PRP adjusted to $3 \times 10^8$ cells $ml^{-1}$ and melanoma cells preincubated for 1 h at 37°C before addition to 0.4 ml of PRP (Boukerche *et al.*, 1989*a*). Melanoma cells ($4 \times 10^6$ cells) were preincubated with saturating concentrations of LYP8 (24 μg $10^{-6}$ cells) or LYP18 (14 μg $10^{-6}$ cells), washed three times with HBSS containing 0.2% BSA then added to PRP. Platelet aggregation by melanoma cells was quantified by measuring the peak height of the aggregation curve as previously described (Boukerche *et al.*, 1989*a*).

*Electron microscopy of platelet-melanoma interaction*

At the end of the aggregation curve, platelet-melanoma suspensions were fixed with 0.15% glutaraldehyde in 0.1 mol $l^{-1}$ cacodylate buffer and then filtered through 0.22 μm filters (Millipore, France). The fixed material was processed for electron microscopy as previously described using a Siemens ELM 102 transmission electron microscope (Boukerche *et al.*, 1989*a*).

*Immunoprecipitation*

Confluent melanoma cells were metabolically labelled with $^{35}S$ for 24 h and chased for 18 h in the absence of labelled methionine. The Triton X-100-extracted melanoma chase media were immunoprecipitated with an anti-TSP MAb (LYP8) or a non-immune mouse serum IgG and analysed by sodium dodecyl sulphate-polyacrylamide gel electrophoresis (SDS-PAGE) as previously described (Boukerche *et al.*, 1989*b*).

*Isolation of thrombospondin and vitronectin receptors*

Thrombospondin (TSP) was purified from the supernatant of ionophore-activated human platelets on a heparin-Sepharose CL-6B column in the presence of 2 mM calcium following the method of Lawler *et al.*, (1988). Vitronectin receptor (VnR) was isolated from melanoma cells by LYP18-affinity chromatography as previously described (Boukerche and McGregor, 1988). The VnR was more than 95% pure when analysed by SDS-PAGE.

*Binding studies*

Human melanoma cells grown to confluence in serum-free RPMI were detached from culture plates by brief treatment with trypsin-EDTA and resuspended in RPMI-1640 containing 0.35% BSA. Thrombospondin (TSP) or isolated antibody was labelled with $^{125}I$ as previously described (Boukerche and McGregor, 1988). Labelled TSP or antibody was separated from free $^{125}I$ on a PD-10 column (Sephadex G-25 M). Increasing concentrations of $^{125}I$-labelled TSP (0.4-24 μg $ml^{-1}$) or $^{125}I$-labelled LYP8 (0.4-24 μg $ml^{-1}$) were added to $10^6$ cells and incubated overnight at 4°C. At the end of the incubation time, aliquots of the melanoma-antibody or melanoma-TSP mixture were layered in triplicate in 400 μl Eppendorf tubes containing 20% sucrose, 2% bovine serum albumin prepared in PBS, pH 7.2. The amount for $^{125}I$-labelled TSP or $^{125}I$-labelled LYP8 bound per melanoma cell was determined by counting the radioactivity of the cut-off centrifuge tips. Non-specific binding was measured by incubating cells with a 100-fold excess of unlabelled TSP or monoclonal antibody. This non-specific binding (less than 10-15%) was subtracted from the total binding. The number of binding sites per cell and the dissociation constant were obtained by double-reciprocal plots and least-squares regression analysis. The values reported are the mean of three experiments. Binding of TSP to vitronectin receptor was studied using an ELISA as described previously (Boukerche and McGregor, 1988). Briefly, purified $\alpha_v\beta_3$ (2-3 μg) diluted in PBS, pH 7.2, containing 2 mM calcium chloride was adsorbed on each well of the microtitre plate for overnight incubation at 4°C. Wells were then washed in PBS containing 1 mM calcium chloride supplemented with 0.05% Tween 20, and increasing concentrations of TSP or albumin (0.1-1.5 μg) diluted in PBS containing 2 mM calcium chloride were added to wells for overnight incubation at 4°C. Plastic wells were then washed and anti-TSP polyclonal antibody was added for 1 h at 37°C to detect bound TSP. After additional washes, goat anti-rabbit antibody conjugated to horseradish peroxidase was added and bound anti-TSP polyclonal antibody was quantified by the addition of the substrate orthophenyldiethylamine.

*Quantification by ELISA of secreted thrombospondin by melanoma cells*

Melanoma cells in complete medium were plated at $3.5 \times 10^5$ cells per well and grown for 1 day. The cells were then extensively washed with serum-free RPMI and incubated for 6 h in serum-free RPMI containing 0.2% BSA. Cells were centrifuged at 200 *g* for 10 min at 4°C and TSP secreted into the culture medium was assayed as previously described (Riser *et al.*, 1988) using a TSP ELISA kit (Stago, France).

*Cell adhesion assays*

The cell adhesion assay was performed as previously described (Varani *et al.*, 1986). Briefly, 2-3 μg of TSP or BSA diluted in PBS, pH 7.2, containing 1 mM calcium chloride and 0.5 mM magnesium chloride was adsorbed on each well of the microtitre plate for 1 h at 37°C. After washing, the wells were blocked with serum-free minimum essential medium (MEM) supplemented with 0.2% BSA to minimise non-specific tumour cell adhesion. Anti-TSP MAb (LYP8) was then added to the wells and incubated for 20 min at 37°C. Melanoma cells were briefly harvested from culture by trypsinisation or by replacing the medium with PBS, pH 7.2, containing 2.5 mM EDTA, washed in serum-free MEM and resuspended in serum-free MEM containing 0.2 mg $ml^{-1}$ BSA. Cells ($10^4$) were then added to coated wells for 60–90 min at 37°C. After gently washing, wells were then fixed with glutaraldehyde and stained with 1.5% Giemsa. Attached and spread cells were counted microscopically. In other experiments, cells were preincubated for 20 min at 37°C with anti-$\alpha_v\beta_3$ MAb (LYP18) (14 μg $10^{-6}$) before being added to TSP-coated wells. Cells adhering to albumin-coated wells were not significant (less than 2%) compared with cells adhering to TSP-coated wells.

*Tumorigenicity assays*

Melanoma cells were harvested by trypsinisation and washed three times with serum-containing medium, then resuspended in PBS, pH 7.2. Two hundred microlitres of $M_3Da$. cells ($1 \times 10^6$) were grafted subcutaneously (s.c.), as previously described (Boukerche *et al.*, 1989*b*), on the ventral surface of nude mice, after preincubation for 20 min with purified LYP8 (24 μg $10^{-6}$), LYP18 (7 μg $10^{-6}$), a non-immune mouse serum IgG or $G_7A_5$ (directed against a melanoma cell-surface antigen other than the vitronectin receptor $\alpha_v\beta_3$ or thrombospondin). Tumour sizes at different time points were expressed as the mean of the sum of two perpendicular diameters. In these experiments, five mice were used for each time point.

*Assays for effect of LYP8 on tumour cell proliferation and nucleic acid synthesis*

*Tumour cell proliferation* Aliquots ($6.5 \times 10^4$) of melanoma cell suspension preincubated for 10 min at room temperature with a saturating concentration of MAb LYP8 (24 μg $10^{-6}$) or a non-immune mouse serum IgG were seeded to each of the 96 wells of the flat-bottomed tissue culture plate. At selected time, cells were washed with Dulbecco's PBS, trypsinised and counted. Data are the mean of six well counts.

*Nucleic acid synthesis* Aliquots ($15 \times 10^3$) of melanoma cell suspension preincubated with saturating concentration of MAb LYP8 (24 μg $10^{-6}$) or a non-immune mouse serum IgG were added to each of the 24 wells of the flat-bottomed tissue culture plate and cultured for 1–2 days. The cells in each well were pulsed for 12 h with [$^3$H]thymidine (0.0185 MBq per well, 37 GBq $mmol^{-1}$, Amersham). Cells were then washed with Dulbecco's PBS, trypsinised and radioactivity incorporated in the cells counted. Data are the mean of 12 well counts.

### Results

*Cell-surface expression of thrombospondin (TSP) by $M_3Da$.*

An ELISA, performed on the conditioned media of cultured cells, showed that $M_3Da$. secreted 80 ± 6 ng TSP $10^{-6}$ cells (mean ± s.d., $n = 3$). LYP8, a monoclonal antibody (MAb) directed against platelet TSP and a potent inhibitor of platelet aggregation induced by thrombin and collagen (Boukerche and McGregor, 1988), immunoprecipitated from chase media of metabolically labelled $M_3Da$. melanoma cells a protein having the same apparent molecular weight (mol. wt) as TSP with the characteristic changes in mobility on reduction (Figure 1a). This MAb was observed by immunofluorescence staining to bind to the surface of $M_3Da$. melanoma cells (Figure 1b). This staining appeared uniformly distributed over the entire surface of $M_3Da$. cells, with high fluorescence intensity in some areas of the membrane, suggesting the presence of clusters of TSP. Alternatively, this clustering may be an artefact due to multivalent interaction of the first antibody with cell-surface TSP. Similarly, as previously reported, $M_3Da$. cells stained with an anti-$\alpha_v\beta_3$ MAb LYP18 (Figure 1b) (Boukerche *et al.*, 1989*b*). In contrast, an anti-CD36, polyclonal or MAb OKM5, did not bind to $M_3Da$. cells (Figure 1b). To determine the number of TSP molecules expressed on the surface of melanoma cells, binding studies using $^{125}$I-labelled LYP8 were performed. LYP8 binding to $M_3Da$. melanoma cells was specific, concentration-dependent and saturable with 367 000 ± 58 000 (mean ± s.d., $n = 3$) LYP8 binding sites per cell and a dissociation constant ($K_d$) of 67 nM (Figure 2). Binding of labelled LYP8 to $M_3Da$. cells was reduced by 90% in the presence of a 100-fold excess of unlabelled TSP. $^{125}$I-labelled TSP binding to $M_3Da$. cells was concentration dependent over the range of 0.4 to 24 μg $10^{-6}$ with at saturation 400 000 ± 50 000 TSP binding sites per cell (mean ± s.d., $n = 3$) and a dissociation constant ($K_d$) of 10 nM. In the presence of an excess of cold TSP, binding of $^{125}$I-labelled TSP was reduced to less than 85%. LYP8 (10 μg $ml^{-1}$) did not affect the binding of labelled TSP to $M_3Da$. cells or to platelets stimulated with thrombin (0.4 U $ml^{-1}$) (results not shown). In human blood platelets and in certain tumour cell lines, CD36 was shown to act as one of the TSP receptors (Asch *et al.*, 1987; McGregor *et al.*, 1989; Silverstein *et al.*, 1992). These results clearly demonstrate that $M_3Da$. synthesise and secrete into the culture medium a protein immunologically related to TSP that binds to the cell surface via receptors different from CD36.

*Inhibition of platelet–melanoma interaction by anti-thrombospondin (TSP) MAb LYP8*

Since $M_3Da$. cells synthesise TSP and bind LYP8, the role of TSP in tumour–platelet interaction was investigated. $M_3Da$. cells irreversibly aggregated human platelets in heparinised PRP (Figure 3). Platelets washed by the technique of Mustard *et al.* (1972) are not aggregated by $M_3Da$. melanoma cells (results not shown). Addition to platelets of LYP8 (24 μg $10^{-6}$ cells) inhibited platelet aggregation induced by $M_3Da$. (Figure 3). If $M_3Da$. cells are preincubated with a saturating concentration of LYP8 (24 μg $10^{-6}$ cells), washed three times with HBSS–BSA, then added to platelets, aggregation is blocked (Figure 3). Anti-TSP MAbs, LYP10 or LYP12, used at the same concentration or a non-immune mouse serum IgG, had no effect on platelet aggregation induced by melanoma cells. As previously shown, an anti-$\alpha_v\beta_3$ MAb (LYP18) added to $M_3Da$. cells significantly inhibited platelet aggregation induced by $M_3Da$. cells (Figure 3) (Boukerche *et al.*, 1989*a*).

*Electron microscopy of melanoma–platelet interactions*

Electron microscopy studies showed that melanoma cells closely interacted with platelets (Figure 4a). Melanoma cells at the site of their interaction with platelets formed extrusions or processes which penetrated into the platelet aggregates (Figure 4b) (Boukerche *et al.*, 1989*a*). Addition to a platelet–tumour cell mixture of anti-TSP MAb LYP8 resulted in a significant decrease in the size of platelet aggregates (Figure 4c). Moreover, platelet–melanoma cell interactions could not be observed, confirming the aggregometry results (Figure 4c). Moreover, in the presence of LYP8, the tumour cell surface did not show cytoplasmic extrusion or processes (Figure 4c).

*Effect of LYP8 on melanoma tumour growth in nude mice*

The biological role of TSP binding to $M_3Da$. cells was investigated by implanting subcutaneously the $M_3Da$. cell line in nude mice and following the size and time of *in vivo* tumour growth over a period of 6 weeks. In the presence of LYP8 (24 μg $10^{-6}$ cells), $M_3Da$. cells are inhibited from growing into full-sized tumours as observed in control animals (Figure 5a). Similar inhibition was observed with an anti-$\alpha_v\beta_3$

**Figure 1** (**a**) Immunoprecipitation by LYP8 monoclonal antibody (MAb) of thrombospondin (TSP) from chase medium of metabolically labelled ([$^{35}$S]methionine) $M_3$Da. ( = $M_3$Dau.) human melanoma cells. Immunoprecipitates were applied to 5–15% exponential gradient SDS–polyacrylamide gels and electrophoresed under non-reducing (NR) or reducing (R) conditions. Lanes A. chase medium; lanes B and E, immunoprecipitates with LYP8 MAb; lanes C and D, immunoprecipitates with non-immune mouse serum IgG. (**b**) Immunofluorescence staining of $M_3$Da. human melanoma cells by an anti-TSP MAb LYP8. $M_3$Da. cells were incubated with LYP8 or an anti $\alpha_v\beta_3$ MAb (LYP18) or an anti-CD36 MAb (OKM5). Rabbit anti-mouse IgG F(ab')$_2$ antibody conjugated to fluorescein isothiocyanate (FITC) was then added to cells.

MAb LYP18 (Figure 5a). The inhibition of tumour cell growth by LYP8 or LYP18 extends over a period of 40 days. Over that period $M_3$Da. cells gave rise to small tumours growing at a lower rate than in the controls. Other anti-TSP MAbs (LYP10, LYP12, directed respectively against the 70 kDa trypsin-resistant core region and the heparin-binding domain of TSP) had no effect on tumour growth. Combination of the two MAbs (LYP8 + LYP18), used at saturating concentrations, gave similar results as that obtained with anti-$\alpha_v\beta_3$ MAb LYP18. To rule out the possibility that LYP8 might have a direct cytotoxic effect, melanoma cells were preincubated with excess LYP8 (24 $\mu$g ml$^{-1}$) and monitored for cell viability and cell growth. No loss of melanoma cells viability was shown by trypan blue exclusion. Furthermore, control and LYP8-treated cells showed a similar degree of [$^3$H]thymidine uptake and cell growth *in vitro* (Figure 5b and c). The lack of effect caused by the antimelanoma MAb $G_7A_5$ suggests that the observed inhibition by LYP8 or LYP18 *in vivo* was not due to complement-dependent cytotoxicity or opsonisation. Moreover, in the presence of fresh rabbit or nude mice sera containing complement, LYP8 did not support lysis of melanoma cells (results not shown). As previously reported, natural killer cells are not involved in $M_3$Da. growth in nude mice (Jacubovich *et al.*, 1984).

### *Adhesion assays*

In order to study the mechanism allowing an anti-TSP MAb (LYP8) to inhibit tumour growth, we looked at the ability of TSP to support $M_3$Da. adhesion, and the effect of LYP8 on such an interaction. $M_3$Da. cells attached to TSP-coated wells with 20% of cells spreading (Figure 6a). LYP8 had no effect on the attachment of $M_3$Da. cells to TSP but decreased significantly the number of cells spreading on TSP (Figure 6a and b). Extending the incubation time of tumour cell adhesion on TSP with LYP8 from 60 to 90 min gave similar results (results not shown). TSP secretion does not promote shedding of antibody since $^{125}$I-labelled LYP8 bound to TSP-

**Figure 2** Binding of $^{125}$I-labelled anti-thrombospondin (TSP) monoclonal antibody (MAb) (LYP8) to $M_3$Da. human melanoma cells. Human melanoma cells grown to confluence in serum-free RPMI were detached by trypsin-EDTA or EDTA and resuspended in RPMI-1640 containing 0.35% BSA. Increasing concentrations of $^{125}$I-labelled LYP8 were added and incubated overnight at 4°C. The insert shows a double-reciprocal plot of the same data with $K_d = 66.6$ nM and the maximum number of binding sites of $4 \times 10^5$ per cell. Non-specific binding of labelled LYP8 was obtained by using a 100-fold excess of unlabelled LYP8.

**Figure 3** Effect of an anti-thrombospondin (TSP) monoclonal antibody (MAb) (LYP8) on aggregation of platelets in PRP induced by $M_3$Da. ( = $M_3$Dau.) human melanoma cells. Typical aggregation curves of platelet in heparinised PRP induced by the addition of $M_3$Da. melanoma cells ($4 \times 10^6$). Controls were either HBSS-BSA buffer or a non-immune mouse serum IgG. LYP8 (24 μg $10^{-6}$) or LYP18 (14 μg $10^{-6}$) were added to platelets before the addition of $M_3$Da. melanoma cells. Similar results were obtained with $M_3$Da. melanoma cells pretreated with LYP8 (24 μg $10^{-6}$), washed three times with HBSS-BSA, then added to platelets.

**Figure 4** Ultrastructural analysis of $M_3$Da. human melanoma-platelet interactions. At the end of the aggregation curve, platelet-melanoma suspensions were fixed with glutaraldehyde and prepared for electronmicroscopy as described in Materials and methods. (**a**) Platelets aggregates (p) in direct contact with $M_3$Da. melanoma cells (m) ( × 3400). (**b**) At higher magnification, melanoma (m) showed extrusions (arrows) at the site of their interaction with platelets (p) ( × 12 500). (**c**) Platelets (p) not interacting with $M_3$Da. melanoma cells (m) when platelets were preincubated with LYP8 ( × 3400). Bar = 2 μm.

**Figure 5** (**a**) Inhibition of melanoma tumour growth in nude mice by anti-thrombospondin (TSP) monoclonal antibody (MAb) (LYP8). $M_3Da$. cells ($1 \times 10^6$) were grafted subcutaneously (s.c.) on the ventral face of nude mice after preincubation for 10 min with purified LYP8 ($24\,\mu g\,10^{-6}$) or anti-$\alpha_v\beta_3$ MAb LYP18 ($14\,\mu g\,10^{-6}$) or a combination of the two MAbs used at saturating concentration. Controls were non-immune mouse serum IgG or MAb $G_7A_5$ directed against a melanoma cell-surface antigen. (**b**) Effect of anti-thrombospondin (TSP) monoclonal antibody (MAb) (LYP8) on *in vitro* tumour cell proliferation. (**b**) $M_3Da$. cells ($6.5 \times 10^5$ per well) preincubated with a non-immune mouse serum IgG or MAb LYP8 ($24\,\mu g\,10^{-6}$) were cultured in RPMI medium containing 10% fetal calf serum. At selected times, viable tumour cells were counted with a haemocytometer. Data are the mean of six well counts. (**c**) Effect of anti-thrombospondin (TSP) monoclonal antibody (MAb) LYP8 on *in vitro* DNA synthesis. $M_3Da$. cells ($15 \times 10^3$ per well) preincubated with a non-immune mouse serum IgG or MAb LYP8 ($24\,\mu g\,10^{-6}$) were cultured for 1–2 days in RPMI medium containing 10% fetal calf serum. The cells were then pulsed with [$^3$H]thymidine for 12 h. Radioactivity incorporated in the cells was measured by a standard liquid scintillation counting. Data are the mean of 12 well counts.

**Figure 6** (**a**) Phase-contrast photomicrographs of $M_3Da$. cell attachment and spreading on TSP in the presence of anti-TSP monoclonal antibody (MAb) LYP8. Wells were coated with TSP (2–3 μg) and adhesion assays were performed as described in Materials and methods in the presence of MAb LYP8 or a control non-immune mouse serum IgG. Cells were fixed with glutaraldehyde and photographed at a final magnification of 100 ×. Cells spread on TSP are indicated by arrows. (**b**) Effect of anti-thrombospondin (TSP) monoclonal antibody (MAb) (LYP8) or anti-$\alpha_v\beta_3$ MAb (LYP18) on $M_3Da$. spreading on TSP. Cells fixed with glutaraldehyde and counted microscopically. Data are expressed as the mean ± s.d., $n = 3$.

coated wells in the presence of $M_3Da$. cells was not released into the supernatant (less than 1% of the total TSP bound LYP8 was recovered in the supernatant). Similarly, an anti-$\alpha_v\beta_3$ MAb, LYP18, had no effect on attachment but inhibited spreading of $M_3Da$. cells on TSP (Figure 6b). To examine further the specificity of TSP binding to $\alpha_v\beta_3$, an ELISA was performed. Preliminary experiments performed with Triton X-100 melanoma cell lysate using LYP18 and LYP8 in a double-antibody sandwich ELISA showed that TSP binds to $\alpha_v\beta_3$ (results not shown). Further experiments were then performed with purified $\alpha_v\beta_3$. Purified $\alpha_v\beta_3$ was added to wells of the microtitre plate followed by the addition of increasing concentrations of TSP or albumin. Binding of TSP to $\alpha_v\beta_3$ bound to LYP18 was significant compared with albumin (Figure 7). These results show that TSP in $M_3Da$. interacts with $\alpha_v\beta_3$ receptors and exclude the possibility that the observed binding may be due to minor contaminants present in the sample.

### Discussion

This study indicates that one of the adhesive ligands playing a key role in human melanoma ($M_3Da$.) cell interaction with platelets is thrombospondin (TSP). Moreover, TSP also appears to play a crucial role in the control of $M_3Da$. tumour growth. The site on TSP to which monoclonal antibody (MAb) LYP8 is directed appears, in contrast to other anti-TSP MAb's (LYP10, LYP12) binding to different epitopes, to play an important role in melanoma–platelet interaction and tumour growth. Several lines of evidence back the above statements:

(1) TSP binds with a high affinity to the surface of $M_3Da$. via receptors that differ from CD36, not expressed by $M_3Da$.

**Figure 7** ELISA detection of thrombospondin (TSP)-$\alpha_v\beta_3$ complex formation. Purified $\alpha_v\beta_3$ (1-2 μg) was coated on the wells as described in Materials and methods. After incubation and washing, increasing concentrations of TSP or albumin (0.1-1.5 μg) were added to the wells and bound TSP was probed with anti-TSP polyclonal antibody followed by horseradish peroxidase conjugated to goat anti-rabbit antibody. Binding of anti-TSP polyclonal antibody was quantified by the addition of the substrate orthophenyldiethylamine. Data are the mean of three experiments.

(2) Reducing the availability or functional level of TSP by using an anti-TSP MAb (LYP8) resulted in a significant decrease of platelet aggregates interacting with melanoma.
(3) Tumour formation *in vivo* was also affected by the presence of bound LYP8.
(4) LYP8 and an anti $\alpha_v\beta_3$ (LYP18) inhibited in a similar way spreading of $M_3Da$. cells to coated TSP.
(5) Purified $\alpha_v\beta_3$ binds TSP in an ELISA.

An important point to be added to the above observations is that Nak$^{a-}$ platelets deficient in CD36 have been shown to aggregate normally in the presence of $M_3Da$. melanoma cells (H Boukerche, B Kehrel and JL McGregor, unpublished observations) (Kehrel *et al.*, 1993). The absence of CD36 expression on $M_3Da$. suggests that TSP binds to this melanoma cell line via another receptor. An obvious candidate to bind TSP could be $\alpha_v\beta_3$, which is known to bind to the RGDA sequence of TSP on endothelial and melanoma cells (Lawler *et al.*, 1988; Tuszynski *et al.*, 1989). This receptor ($\alpha_v\beta_3$) has been shown to be expressed by $M_3Da$. and plays, as previously indicated, a crucial role in melanoma-platelet interaction and *in vivo* melanoma tumour growth (Boukerche *et al.*, 1989*a*,*b*; Marshall *et al.*, 1991; Felding-Habermann *et al.*, 1992). Results in this study and previous work (Lawler *et al.*, 1988; Tuszynski *et al.*, 1989) suggest that TSP binding to $\alpha_v\beta_3$, expressed by $M_3Da$., is directly implicated in melanoma-platelet interaction and tumour growth formation *in vivo*. Our data do not exclude a possible role of CD36 present in the microenvironment of a tumour *in vivo*. Results in this study extend observations made by Tuszynski *et al.* (1987, 1992), who showed that whole TSP and a peptide (CSVTCG) derived from this adhesive ligand affect tumour metastasis of mouse melanoma. Moreover, CSVTCG and its analogue have been shown to be potent inhibitors of platelet aggregation (Byck and McGregor, 1992). The recent discovery of four homologous forms of TSP (TSP-1, TSP-2, TSP-3 and TSP-4) encoded by distinct genes indicates that further characterisation of TSP expressed by melanomas is required (Bornstein, 1992).

An anti-TSP MAb (LYP8) significantly affects platelet aggregates interacting with melanoma cells, as shown in the electron micrograph presented in this study. However, LYP8 did not reduce TSP binding to $M_3Da$. cells, nor did it affect TSP binding to washed platelets stimulated with thrombin. These results indicate that LYP8 may interfere by steric hindrance with a mechanism involved in linking tumour cells and platelets together and suggest that TSP coating $M_3Da$. cells is directly involved in mediating tumour cell-platelet interactions. A similar observation was reported with LYP8 and other MAbs directed against GPIIb and IIIa, which together inhibited platelet aggregation without affecting fibrinogen binding (Newman *et al.*, 1987; Boukerche and McGregor, 1988). These results suggest that additional post-TSP binding events such as conformational changes and/or clustering of cell-surface molecules may be required to support tumour-platelet interaction (Peerschke and Zucker, 1981).

Such a role of TSP in mediating cell-cell interactions was also shown with thrombin-activated platelets binding to a monocytic cell line (U937) or to melanoma cells (Nierodzik *et al.*, 1991; Silverstein *et al.*, 1992). In contrast to these findings, platelets in our system are activated by ADP and released by $M_3Da$. cells, under conditions in which presumably no secretion from platelets takes place (Boukerche *et al.*, 1989*a*). Recent results from our laboratory indicate that ADP is present in appreciable amounts (180 ± 10 pmol $10^{-6}$ cells) in the cell supernatant of $M_3Da$. cells as shown by high-performance liquid chromatography (HPLC) (Boukerche *et al.*, 1994). Previous observations have shown that platelets activated by ADP in the presence of physiological levels of $Ca^{2+}$, as present in heparinised PRP, aggregate but do not release their α-granule content (Mustard *et al.*, 1972). Under these conditions, TSP released by $M_3Da$. cells will not bind to platelets that have not undergone release (Leung, 1984; Boukerche and McGregor, 1988). In view of these results, it appears that ADP-activated platelets need to interact with melanoma cell-surface receptors presumably via $\alpha_{IIb}\beta_3$, to undergo complete degranulation as observed in electron-micrographs obtained in this study and in a previous work (Boukerche *et al.*, 1989*a*). Platelet-melanoma interactions may therefore be initiated by : (1) the release of ADP by $M_3Da$. cells and (2) mechanical stimulation induced by the $M_3Da$. cell surface. Melanoma cells, by interacting with the platelet surface, may promote agonist and ADP release from platelets, leading to degranulation and formation of larger platelet-tumour aggregates. TSP coating of degranulated platelet is known to help in cementing platelet aggregates (Leung, 1984, Boukerche and McGregor, 1988). The ability of tumour cells to induce platelet activation *in vitro* has been used as indirect evidence to show the role of platelets in the dissemination of tumour cells (Gasic *et al.*, 1973). TSP linking the tumour-platelet aggregates may provide a vehicle for transport and dissemination of tumour cells. Experiments are under way to determine the metastatic capacity of $M_3Da$. binding TSP.

Tumour growth is the result of a complex interaction between tumour cells and the basement membrane extracellular matrix components (Dvorak *et al.*, 1991). TSP appears to play a crucial role in the control of tumour growth. The finding that anti-TSP MAb LYP8 did not completely inhibit tumour growth suggests that other glycoprotein receptors and adhesive proteins contribute to the full expression of the tumorigenic phenotype of the cells. Adhesive proteins such as TSP binding to melanoma cells may *in vivo* modulate cellular proliferation as previously shown for normal and transformed cells (Majack *et al.*, 1988; Abbadia *et al.*, 1993). Alternatively, MAb LYP8 binding to TSP may block critical interactions between melanoma cells and stromal matrices that are vital for successful angiogenesis (Tolsma *et al.*, 1993; Dameron *et al.*, 1994).

TSP is a multidomain glycoprotein that binds to a number of adhesive receptors (Asch *et al.*, 1987; Lawler *et al.*, 1988; Roberts, 1988; Karczewski *et al.*, 1989; McGregor *et al.*, 1989; Yabkowitz and Dixit, 1991). One of its receptors, CD36, which binds to the 68 kDA TSP fragment via the type I repeat (CSVTCG) in the absence of $Ca^{2+}$, is not expressed by $M_3Da$. cells (Asch *et al.*, 1992; Catimel *et al.*, 1992). In the current study, $M_3Da$. melanoma cells that lack CD36 attached and spread on TSP in a similar way as reported by Roberts *et al.* (1987) for melanoma G361. LYP8 and LYP18 MAbs inhibited $M_3Da$. spreading on TSP. Purified $\alpha_v\beta_3$ was shown to bind TSP. These results and previous work (Lawler et al., 1988; Tuszynski et al., 1989) suggest that TSP interacts with $\alpha_v\beta_3$. Inhibition by LYP8 of TSP-mediated $M_3Da$. spreading and melanoma-platelet interaction could be the result, as previously suggested in this study, of steric or conformational changes of TSP induced by LYP8 (Dixit et al., 1986). Our data do not exclude the possibility that, in addition to $\alpha_v\beta_3$, TSP may bind to other receptors (i.e. heparan sulphate proteoglycans) in view of the lack of effect of LYP8 in inhibiting cell attachment of $M_3Da$. to TSP-coated wells (Roberts, 1988; Asch et al., 1991).

Our results are consistent with recent studies of Castle et al. (1991) showing the importance of TSP by transfecting human squamous carcinoma cells with the TSP cDNA antisense expression vector and decreasing the tumorigenic phenotype of these cells. Moreover, the CSVTCG TSP peptide and its analogue was shown to block cell adhesion, platelet aggregation and tumour cell metastasis (Tuszynski et al., 1992; Byck and McGregor, 1992). Tumour metastasis is a complex sequence of events in which malignant cells enter the bloodstream and interact with various host cells, including vascular endothelial cells, before extravasating and forming secondary tumours (Nicolson, 1988). Reagents such as MAbs against TSP and peptides derived from TSP could not only be used as a new therapeutic approach in the control of tumour metastasis of malignant melanoma, but may also contribute to the elucidation on the role of TSP in cancer biology.

**Acknowledgments**

This work was supported by grants from the Association pour la Recherche contre le Cancer (subvention 6586), the Ligue National Contre le Cancer and Fédération Nationale des Groupements des Enterprises Françaises dans la Lutte Contre le Cancer. We would like to thank Martine Mesh and Monique Groleas for excellent technical assistance.

**References**

ABBADIA Z, AMIRAL J, TRZECIAK M-C, DELMAS PD AND CLEZARDIN P. (1993). Thrombospondin (TSP-1) modulates in vitro proliferation of human MG-63 osteoblastic cells induced by alpha-thrombin. *FEBS Lett.*, **329**, 341-346.

ASCH AS, BARNWELL J, SILVERSTEIN RL AND NACHMAN RL. (1987). Isolation of thrombospondin membrane receptor. *J. Clin. Invest.*, **79**, 1054-1057.

ASCH AS, TEPLER J, SILBIGER S AND NACHMAN RL. (1991). Cellular attachment to thrombospondin. Cooperative interactions between receptor systems. *J. Biol. Chem.*, **266**, 1740-1745.

ASCH AS, SILBIGER S, HEIMER E AND NACHMAN RL. (1992). Thrombospondin sequence motif (CSVTCG) is responsible for CD36 binding. *Biochem. Biophys. Res. Commun.*, **182**, 1208-1217.

BORNSTEIN P. (1992). Thrombospondins:structure and regulation of expression. *FASEB J.*, **6**, 3290-3299.

BOUKERCHE H AND McGREGOR JL. (1988). Characterization of an anti-thrombospondin monoclonal antibody (P8) that inhibits human blood platelet functions. Normal binding of P8 to thrombin-activated Glanzmann thrombasthenic platelets. *Eur. J. Biochem.*, **171**, 383-388.

BOUKERCHE H, BERTHIER-VERGNES O, TABONE E, DORE J-F, LEUNG LLK AND McGREGOR JL. (1989*a*). Platelet-melanoma interaction is mediated by the glycoprotein IIb-IIIa complex. *Blood*, **74**, 658-663.

BOUKERCHE H, BERTHIER-VERGNES O, BAILLY M, DORE J-F, LEUNG LLK AND McGREGOR JL. (1989*b*). A monoclonal antibody (LYP18) directed against the blood platelet glycoprotein IIb-IIIa complex inhibits human melanoma growth in vivo. *Blood*, **74**, 909-912.

BOUKERCHE H, BERTHIER-VERGNES O, PENIN F, TABONE E, LIZARD G, BAILLY M AND McGREGOR JL. (1994). Human melanoma cell lines differ in their capacity to release ADP and aggregate platelets. *Br. J. Haematol.*, **87**, 763-772.

BYCK G AND McGREGOR JL. (1992). Peptide analogs derived from the thrombospondin type I repeat (CSVTCG) inhibit platelet aggregation. In *Peptides*, Schneider CH and Eberle AN. (eds) pp. 827-828. Escom Science Publisher: Leiden.

CATIMEL B, LEUNG LLK, EL GHISSASI H, MERCIER N AND McGREGOR JL. (1992). Human platelet glycoprotein IIIb binds to thrombospondin fragments bearing the C-terminal region, and/or the type I repeats (CSVTCG motif), but not the N-terminal heparin-binding domain. *Biochem.* **284**, 213-236.

CASTLE V, VARANI J, FLIGIEL S, PROCHOWNICK EV AND DIXIT V. (1991). Antisense-mediated reduction in TSP reverses the malignant phenotype of a human squamous carcinoma. *J. Clin. Invest.*, **87**, 1883-1888.

DAMERON KM, VOLPERT OV, TAINSKY MA AND BOUCK N. (1994). Control of angiogenesis in fibroblasts by p53 regulation of thrombospondin-1. *Science*, **265**, 1582-1584.

DIXIT VM, O'ROURKE KM, GRANT GA, SANTORO SA AND FRAZIER. (1986). Monoclonal antibodies that recognize calcium-dependent structures of human thrombospondin with EM and high sensitivity amino acid sequencing. *J. Biol. Chem.*, **261**, 1962-1968.

DVORAK HF, NAGY JA AND DVORAK AM. (1991). Structure of solid tumors and their vasculature: implications for therapy with monoclonal antibodies. *Cancer Cells*, **3**, 77-85.

FELDING-HABERMANN B, MUELLER BM, ROMERDAHL CA AND CHERESH D. (1992). Involvement of $\alpha_v$ integrin gene expression in human melanoma tumorigenecity. *J. Clin. Invest.*, **89**, 2018-2022.

GASIC GJ, GASIC TB, GALANTI N, JOHNSON T AND MURPHY S. (1973). Platelet-tumour cell interactions in mice. The role of platelets in the spread of malignant disease. *Int. J. Cancer*, **11**, 704-718.

JACUBOVITCH R, CABRILLAT H AND DORE J-F. (1984). Natural resistance to xenografts of human malignant melanoma cell lines in nude mice. *Exp. Cell Biol.*, **52**, 48-52.

KARCZEWSKI JKA, KNUDSEN KA, SMITH L, MURPHY A, ROTHMAN VL AND TUSZYNSKI GP. (1989). The interaction of thrombospondin with platelet glycoprotein GPIIb-IIIa. *J. Biol. Chem.*, **264**, 21322-21326.

KEHREL B, KRONENBERG A, RAUTERBERG J, NIESING-BRESCH D, NIEHUES U, KARDOEUS J, SCHWPPERT B, TSCHÖPE D, VANDE LOO J AND CLEMETSON KJ. (1993). Platelets deficient in glycoprotein IIIb aggregate normally to collagen type I and III but not to collagen type V. *Blood*, **82**, 3364-3370.

LAWLER J. (1986). The structure and functional properties of TSP. *Blood*, **67**, 1197-1209.

LAWLER J, WEINSTEIN R AND HYNES RO. (1988). Cell attachment to thrombospondin: the role of Arg-Gly-Asp., calcium and integrin receptors. *J. Cell. Biol.*, **107**, 2351-2361.

LEUNG LLK. (1984). The role of thrombospondin in platelet aggregation. *J. Clin. Invest.*, **74**, 1764-1772.

McGREGOR JL AND BOUKERCHE H. (1993). Thrombospondin interaction with human blood platelets. In *Thrombospondin*, Lahav J. (ed.) pp. 111-127. CRC Press: New York.

McGREGOR JL, CATIMEL B, PARMENTIER S, CLEZARDIN P, DECHAVANNE M AND LEUNG LLK. (1989). Rapid purification and characterization of human platelet glycoprotein IIIb. *J. Biol. Chem.*, **264**, 501-506.

MAJACK RA, GOODMAN LV AND DIXIT VM. (1988). Cell surface TSP is functionally essential for vascular smooth muscle cell proliferation. *J. Cell. Biol.*, **106**, 415-422.

MARSHALL JF, NESBITT SA, HELFRICH MH, HORTON MA, POLAKOVA K AND HART, IR. (1991). Integrin expression in human melanoma cell lines: heterogeneity of vitronectin receptor composition and function. *Int. J. Cancer*, **49**, 924-931.

MUSTARD JF, PERRY DW, ARDLIE NG AND PACKHAM M. (1972). Preparation of suspensions of washed platelets from humans. *Br. J. Haematol.*, **22**, 193-204.

NEWMAN PJ, MCEVER RP, DOERS MP AND KUNICKI TJ. (1987). Synergistic action of two murine monoclonal antibodies that inhibit ADP-induced platelet aggregation without blocking fibrinogen binding. *Blood*, **69**, 668-676.

NICOLSON GL. (1988). Cancer metastasis: tumour cell and host organ properties important in metastasis to specific secondary sites. *Biochim. Biophys. Acta*, **948**, 175-224.

NIERODZIK ML, PLOTKIN A, KAJUMO F AND KARPATKIN S. (1991). Thrombin stimulates tumour-platelet adhesion *in vitro* and metastases *in vivo*. *J. Clin. Invest.*, **87**, 229-236.

PEERSCHKE EJ AND ZUCKER MB. (1981). Fibrinogen receptor and aggregation of human blood platelets produced by ADP and chilling. *Blood*, **57**, 663-70.

RISER BL, VARANI J, O'ROURKE K AND DIXIT VM. (1988). Thrombospondin binding by human squamous carcinoma and melanoma cells. Relationship to biological activity. *Exp. Cell Res.*, **174**, 319-329.

ROBERTS DD. (1988). Interactions of thrombospondin with sulfated glycolipids and proteoglycans of human melanoma cells. *Cancer Res.*, **48**, 6785-6793.

ROBERTS DD, SHERWOOD JA AND GINSBURG V. (1987). Platelet thrombospondin mediates attachment and spreading of human melanoma cells. *J. Cell. Biol.*, **104**, 131-139.

SILVERSTEIN RL, BAIRD M, KONG LO S AND YESNER LM. (1992). Sense and antisense cDNA transfection of CD36 (glycoprotein IV) in melanoma cells. Role of CD36 as a thrombospondin receptor. *J. Biol. Chem.*, **267**, 16607-16612.

TOLSMA SS, VOLPERT OV, GOOD DJ, FRAZIER WA, POLVERINI PJ AND BOUCK N. (1993). Peptides derived from two separate domains of the matrix protein thrombospondin -1 have anti-angiogenic activity. *J. Cell Biol.*, **122**, 497-511.

TUSZYNSKI GP AND KOWALSKA MA. (1991). Thrombospondin-induced adhesion of human platelets. *J. Clin. Invest.*, **87**, 1387-1394.

TUSZYNSKI GP, GASIC TB, ROTHMAN VL, KNUDSEN KA AND GASIC GJ. (1987). Thrombospondin, a potentiator of tumor cell metastasis. *Cancer Res.*, **47**, 4130-4133.

TUSZYNSKI GP, KARCZEWSKI J, SMITH L, MURPHY L, ROTHMAN VL AND KNUDSEN KA. (1989). The GPIIb-IIIa-like complex may function a human melanoma cell adhesion receptor for thrombospondin. *Exp. Cell Res.*, **182**, 473-481.

TUSZYNSKI GP, ROTHMAN VL, DEUTCH AH, HAMILTON BK AND EYAL J. (1992). Biological activities of peptides and peptides analogues derived from common sequences present in TSP, properdin and malaria proteins. *J. Cell Biol.*, **116**, 209-217.

VARANI J, DIXIT VM, FLIGIEL SEG, MCKEEVER PE AND CAREYS TE. (1986). Thrombospondin-induced attachment and spreading of human squamous carcinoma cells. *Exp. Cell. Res.*, **167**, 376-390.

VARANI J, RISER BL, HUGHES LA, CAREYS TE, FLIGIEL SEG AND DIXIT VM. (1989). Characterization of thrombospondin synthesis, secretion and cell surface expression by human tumor cells. *Clin. Exp. Metast.* **7**, 265-276.

WALZ DA. (1992). Thrombospondin as a mediator of cancer cell adhesion and metastasis. *Cancer Metast. Rev.* **11**, 313-324.

YABKOWITZ R AND DIXIT VM. (1991). Human carcinoma cells bind TSP through a Mr. 80,000-105,000 receptor. *Cancer Res.*, **51**, 3648-3656.